# Python algorithm package for automated Estimation of major legume root traits using two dimensional images

**DOI:** 10.1038/s41598-025-91993-y

**Published:** 2025-03-01

**Authors:** Amit Ghimire, Yong Suk Chung, Sungmoon Jeong, Yoonha Kim

**Affiliations:** 1 Department of Applied Biosciences, Kyungpook National University, Daegu, 41566 Republic of Korea; 2 Department of Integrative Biology, Kyungpook National University, Daegu, 41566 Republic of Korea; 3 Department of Plant Resources and Environment, Jeju National University, Jeju, 63243 Republic of Korea; 4 Bio-medical Research Institute, Research Center for AI in Medicine, Kyungpook National University Hospital, Daegu, 41940 Republic of Korea; 5 Department of Medical Informatics, School of Medicine, Kyungpook National University, Daegu, 41566 Republic of Korea; 6 Upland Field Machinery Research Center, Kyungpook National University, Daegu, 41566 Republic of Korea

**Keywords:** Image processing, Legumes, Python algorithm, Root traits, Threshold, Biological techniques, Plant sciences

## Abstract

A simple Python algorithm was used to estimate the four major root traits: total root length (TRL), surface area (SA), average diameter (AD), and root volume (RV) of legumes (adzuki bean, mung bean, cowpea, and soybean) based on two-dimensional images. Four different thresholding methods; Otsu, Gaussian adaptive, mean adaptive and triangle threshold were used to know the effect of thresholding in root trait estimation and to optimize the accuracy of root trait estimation. The results generated by the algorithm applied to 400 legume root images were compared with those generated by two separate software (WinRHIZO and RhizoVision), and the algorithm was validated using ground truth data. Distance transform method was used for estimating SA, AD, and RV and ConnectedComponentsWithStat function for TRL estimation. Among the thresholding methods, Otsu thresholding worked well for distance transform, while triangle threshold was effective for TRL. All the traits showed a high correlation with an R² ≥0.98 (*p < 0.001*) with the ground truth data. The root mean square error (RMSE) and mean bias error (MBE) were also minimal when comparing the algorithm-derived values to the ground truth values, with RMSE and MBE both < 10 for TRL, < 6 for SA, and < 0.5 for AD and RV. This lower value of error metrics indicates smaller differences between the algorithm-derived values and software-derived values. Although the observed error metrics were minimal for both software, the algorithm-derived root traits were closely aligned with those derived from WinRHIZO. We provided a simple Python algorithm for easy estimation of legume root traits where the images can be analyzed without any incurring expenses, and being open source; it can be modified by an expert based on their requirements.

**Supplementary Information:**

The online version contains supplementary material available at 10.1038/s41598-025-91993-y.

## Introduction

Plant phenotyping encompasses the study of complex phenotypic traits, such as growth, shoot and root architecture, yield, tolerance, resistance, and their interactions^1^. Plant phenotyping based on image analysis has always been a topic of research interest and has been used in yield estimation^2–5^, disease detection or identification^6–10^, shoot phenotyping of plants^11–14^, and seed phenotyping^15^. Image processing, which involves the manipulation or evaluation of image properties using existing software or different programming algorithms, plays a pivotal role in this domain^16^. The initial step in image processing is image segmentation, wherein the image is partitioned into different segments with similar texture, intensity, and gray levels^17,18^. Thresholding, a straightforward and effective method for image segmentation, entails evaluating the image based on object and background pixels^18,19^.

Assessment of plant root development through image analysis is a key component of precision agriculture, wherein root images are analyzed to measure root traits, such as surface area (SA), total root length (TRL), average diameter (AD), root volume (RV), number of tips, and number of forks^20,21^. However, examining complex root architecture becomes difficult when dealing with numerous genotypes^12^. Furthermore, root phenotyping poses additional challenges compared with shoot phenotyping due to the roots being situated within the soil^22^. The root system plays a vital role in plant development, offering anchorage, facilitating the supply of water, essential minerals, and nutrients through its unique architecture, and enabling the plant to exploit diverse soil chemical and physical properties^23^. Owing to the complexity of the measurement of root traits (both quantitatively and qualitatively), their utilization as a selection criterion for plant functional analysis is limited^24^. Le Bot et al., (2010) designed software using computer automation known as Data Analysis of Root Tracings that is capable of analyzing global root traits and extracting topological information^20^. Automatic Root Image Analysis (ARIA) software was used to analyze different root traits which involved the process of converting the root images into an equivalent graph^24^. The RhizoChamber software designed by Wu et al., (2018) monitors the growth pattern of the plant’s roots using a robotic platform^5^. Likewise, Woo et al., (2023) estimated the root nodules present in soybean based on deep-learning methods^25^. While some root analysis software is available for free, obtaining results can be time-consuming. Alternatively, high-quality root trait data from other programs often require a substantial purchase cost, hindering the expansion of studies focusing on basic root traits, such as SA, TRL, AD, and RV. In response to these challenges, this study aimed to estimate SA, TRL, AD, and RV based on pixel count using a simple Python algorithm. Being image-based estimation of root traits, this method is designed to be more precise than traditional methods of root trait estimation. There is less need of human intervention as suitable thresholding methods has been tested and used, which makes it more inclined towards precision agriculture. This algorithm is designed to be user-friendly, enabling researchers to estimate root traits without advanced computer knowledge and without incurring any expenses. We employed four different threshold methods in this study: (i) Otsu threshold method: an unsupervised and nonparametric method for automatic thresholding based on variance between clusters determines the optimal image^26,27^. It is the average of the mean levels of two classes divided by this threshold and gives the best threshold value by minimizing the class variance^28,29^. (ii) Triangle threshold method: calculates the threshold value minimizing pixel intensity spread within the image’s foreground and background by connecting two points on the histogram of pixel intensities (typically, the histogram’s endpoints). (iii) Adaptive threshold method: measures spatial variations in the image, smoothing it irrespective of the images used^30,31^. In the case of mean adaptive, it determines the threshold based on the mean value of the neighboring area, while Gaussian adaptive determines the threshold based on the Gaussian-weighted sum of neighborhood values. In addition, various image processing techniques were applied to root images, including conversion to grayscale, median blur for edge smoothing, and inversion of pixel values. The study’s framework is visually presented as a flowchart with corresponding functions in **Fig. ****S1****.**

The novelty of this research and algorithm lies in its ability to estimate the root traits automatically, where we have chosen the best thresholding method so that more accurate results can be obtained. We analyzed the 400 novel root image data sets of four different legumes along with ground truth images. We have provided a comprehensive methodology for measuring each trait and examining the effect of various thresholding methods on root traits, enabling users to adjust it to meet their specific needs. Another important feature of our algorithm is noise reduction, where users can adjust the number of pixel values to reduce noise in their image. There are no special hardware requirements for this advanced software; a computer device with a Python environment is sufficient for analyzing the image. Furthermore, there is no limit to the data size that can be analyzed. As a set of Python algorithms, this package can be used on any operating system (Windows, macOS, Linux, etc.), not confined to a particular operating system. In comparison, programs such as archiDART is an RStudio package that requires additional codes to analyze root images, which can complicate data analysis. Lobet (2017) highlighted that many plant image analysis tools face challenges related to long-term support and validation. Only 62% of these tools remain accessible from their original papers, and approximately 40% are actively maintained. Furthermore, around 29% of the tools lack any validation information regarding their research^32^. This study addresses these shortcomings, aiming to provide a solution for the easy analysis of root traits with enhanced accuracy compared to the high-cost WinRHIZO software. The source code along with the root images and the validation images can be downloaded from (https://github.com/AG9843/Legume-Root-Analysis.git ).

## Materials and methods

### Planting and image acquisition

Seeds of four different types of leguminous crops (soybean, cowpea, mung bean, and adzuki bean) were sown in polyvinyl chloride (PVC) pipes with a diameter of 15 cm and a height of 60 cm in the greenhouse of Kyungpook National University, Daegu, South Korea. Two seeds per pipe were sown and later thinned to a single plant per pipe. In total, 100 plants per legume were maintained, which resulted in 400 leguminous plants. When the second trifoliate leaf emerged (V_2_stage), the plants were harvested. The soil in the pipes was loosened, and the plants were gently taken out along with the roots. The roots were washed with clean tap water to remove any soil debris and packed in plastic bags with some water to prevent drying. The washed roots were then brought to the laboratory for image acquisition. The roots were placed on a transparent plastic tray immersed in water to ensure proper spreading. The tray was scanned using a scanner (Epson, Expression 120000 XL, Japan) to acquire the image. The acquired image was 4414 × 6156 pixels in size with a resolution of 400 dpi, featuring roots in black on a white background. The detailed steps of plant harvest and root image acquisition have also been explained by^33^.

### Programing Language and thresholding

**Fig. 1:**
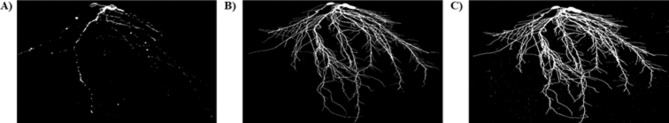
Root images at different threshold level. (**A**) Root image thresholded at 1, 255, (**B**) root image thresholded at 170, 255 and (**C**) root image thresholded at 254, 255.

Python 3.11.4 served as the programming language, and Spyder 5.2.2 was used as the Python working environment for developing the algorithm. The initial step involved the segmentation of scanned root images, where segmentation defines each pixel of the image into two or more classes. In this process, thresholding was employed to divide the image into smaller segments, utilizing at least one color or the grayscale value to delineate the image boundary^34^. Four automated thresholding methods, i.e., triangle, mean adaptive, Gaussian adaptive, and Otsu thresholding, were used for image segmentation. The Otsu threshold method previously used for calculating the surface root area of 2D imagery data of plant roots^35^, was also compared with 15 other methods for analyzing rice root traits^36^. Notably, the triangle method of thresholding, when coupled with a conversion factor of 2/3 for root length determination, yielded optimal results in analysis using ImageJ software^36^. Image thresholding plays an important role in root trait estimation. A lower threshold value erases portions of the root, whereas a much higher value connects neighboring root parts. We have demonstrated the effect of different values of thresholding on the root image when different possible values were used (Fig. 1).

### Trait estimation

#### TRL estimation

**Fig. 2:**
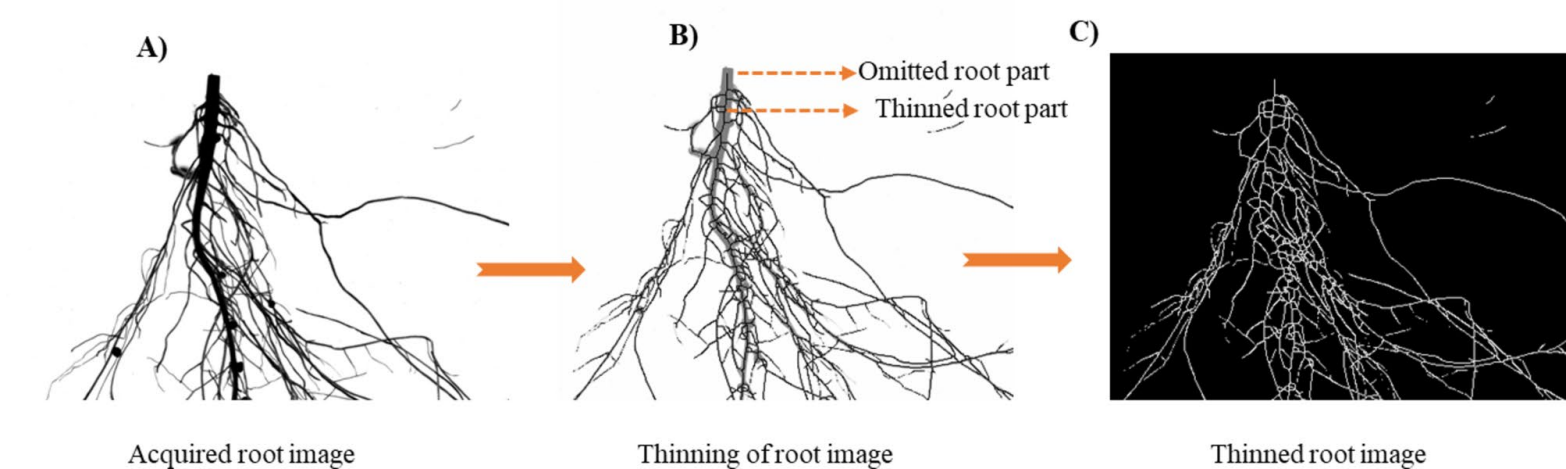
Process of morphological thinning in which the outer root image pixels are removed. A single-pixel root image was acquired, and the number of pixels was counted to give the total length. (**A**) acquired root image, (**B**) thinning of the root image, and (**C**) thinned root image.

In this study, four major root traits, namely, SA, TRL, AD, and RV, were estimated. For TRL estimation, the ConnectedComponentsWithStats function was used to compute the total number of pixels in the root image. The connected components algorithm transforms the threshold or binary image into a symbolic form where all pixels of the same component are assigned the same label^37^. The thresholded image was subjected to thinning to create a single-pixel image. The Ximgproc thinning algorithm was applied to progressively eliminate pixels from the outer edge of the root image until only a single pixel remained, with no further thinning **(**Fig. 2). ConnectedComponentsWithStat function was used to iterate through every pixel that is connected with each other in the skeletal image. After looping through the connected pixel, it was checked whether the connected neighbor pixel was diagonally placed. If the neighboring pixel was connected diagonally, the pixel size was increased by a factor of 1.4142, i.e. (√2). This is the Euclidean distance between the pixels diagonally arranged. If the pixels are connected horizontally or vertically, then the pixel size remains the same, i.e., multiplied by a factor of 1.

#### AD, SA, and RV estimation

Likewise, for measuring SA, AD, and RV distance transform methods through the skeletonized image were used. A distance transform assigns a value to each point in an object, representing the distance from that point to the nearest background point, and this distance is the shortest distance from that point to the background^38^. For AD, the distance transformed through each skeletal pixel was multiplied by the pixel size, which gave the radius (**Equation i**). Multiplying this radius by two gave the diameter (**Equation ii**). Thus, the calculated diameter through each pixel was summed and then divided by the total number of pixels in the skeletal image, which finally gave the AD of the root (**Equation iii**). A similar method was used for the calculation of RV. The cross-sectional area for each skeletal pixel was calculated based on the radius and then summed (**Equation iv**). This total cross-sectional area was then multiplied by pixel size to give the total RV (**Equation v)**. For SA, we calculated the circumference of each skeletal pixel, and then the sum of the total circumference, which, upon multiplication by pixel size, gives the SA of the root (**Equation vi**).

$$\:r=DT\:x\:ps$$ --------------------(i)

$$\:d=2\:x\:r$$ --------------------(ii)

$$\:AD=\frac{d}{N}$$ --------------------(iii)

$$\:CA=\pi\:{r}^{2}$$ --------------------(iv)

$$\:RV=CA\:x\:ps$$ --------------------(v)

$$\:SA=cf\:x\:ps$$ --------------------(vi)

$$\:cf=2\pi\:r$$ ------------------- (vii)

Where ‘*r’* is the radius, ‘*DT’* is the distance transform value, ‘*ps’* is the pixel size, *‘d’* is the total diameter, ‘*AD’* is the average diameter, ‘*N’* is the total number of pixels in the skeletonized image, ‘*CA’* is the cross-sectional area of the specific pixel in the skeletonized image, ‘*RV’* is the root volume, ‘*SA’* is the surface area of the root and ‘*cf’* is the circumference of the specific pixel in the skeletonized image.

#### Traits comparison

The traits derived from our study were evaluated against values obtained from two established software tools: WinRHIZO, developed by Regent Instruments Inc., Canada, and RhizoVision Explorer^39^. The first one is the widely used commercial software, whereas the latter one is freeware. The differences in the software and algorithm method regarding trait estimation has been mentioned in Table 1. The features kept during the analysis of the root using two software has been mentioned in **Table ****S1**. The WinRHIZO calculates two different values for SA and RV. The first type is the estimated values, and the second one is the measured values. The estimated values are based on the trigonometric formula considering roots as cylindrical objects, whereas the measured values are calculated based on the specific trait values for different diameter classes. We compared our results with the measured values of the WinRHIZO. Furthermore, we also compared the WinRHIZO-derived estimated value with the algorithm-derived estimated values and provided the results in Supplementary information. For estimated values following trigonometric formulas were used.

$$\:SA=PA\times\:3\cdot\:141$$ ------------------- (viii)

$$\:AD=\frac{PA}{TRL}$$ ------------------- (ix)

$$\:RV=\pi\:{r}^{2}\:x\:TRL$$ ------------------- (x)

Where ‘*PA’* is the projected area of the root and *‘r*’ is the estimated radius from AD.

**Table 1:** Differences in the software and algorithm method regarding trait estimation.

Traits and methods used	WinRHIZO	RhizoVision	Algorithm
Image processing method	Thresholding	Thresholding	Thresholding
Thresholding method	Both automatic and manual	Semi-automatic (User provided pixel-distance method)	Automatic (Otsu and Triangle)
Imaging device	Scanner, Camera	Scanner, Camera	Scanner
Source code	X	X	Provided
Software package	**✓**	**✓**	Not applicable
Particle clearance	Present	Present	Present
Cost	Paid	Freeware	Free
Modification	Cannot be modified	Cannot be modified	Being bunch of codes can be modified by expert user

### Calibration

The root traits obtained were initially in the form of pixel counts. To convert these pixel counts into a standard unit of measurement (centimeters), we calibrated a ruler. The ruler was scanned using the same settings as the root images, and the Mouse Callback function in OpenCV was used to calibrate the pixels present within a 1-centimeter (cm) span. This was achieved by initially calculating the coordinates between two known points, followed by the calculation of the distance between these coordinates. This distance corresponds to the total number of pixels between the two known points. Another method that can be used is based on the image resolution. The scanned images had a resolution of 400 dpi. The number of pixels in 1 cm^2^ can be obtained by dividing the image resolution by 2.54 (1 inch = 2.54 cm). Therefore, 1 cm^2^ (400/2.54) = 157.48 pixels, and a single pixel size equals 0.0063 cm.

### Validation of the algorithm

**Fig. 3:**
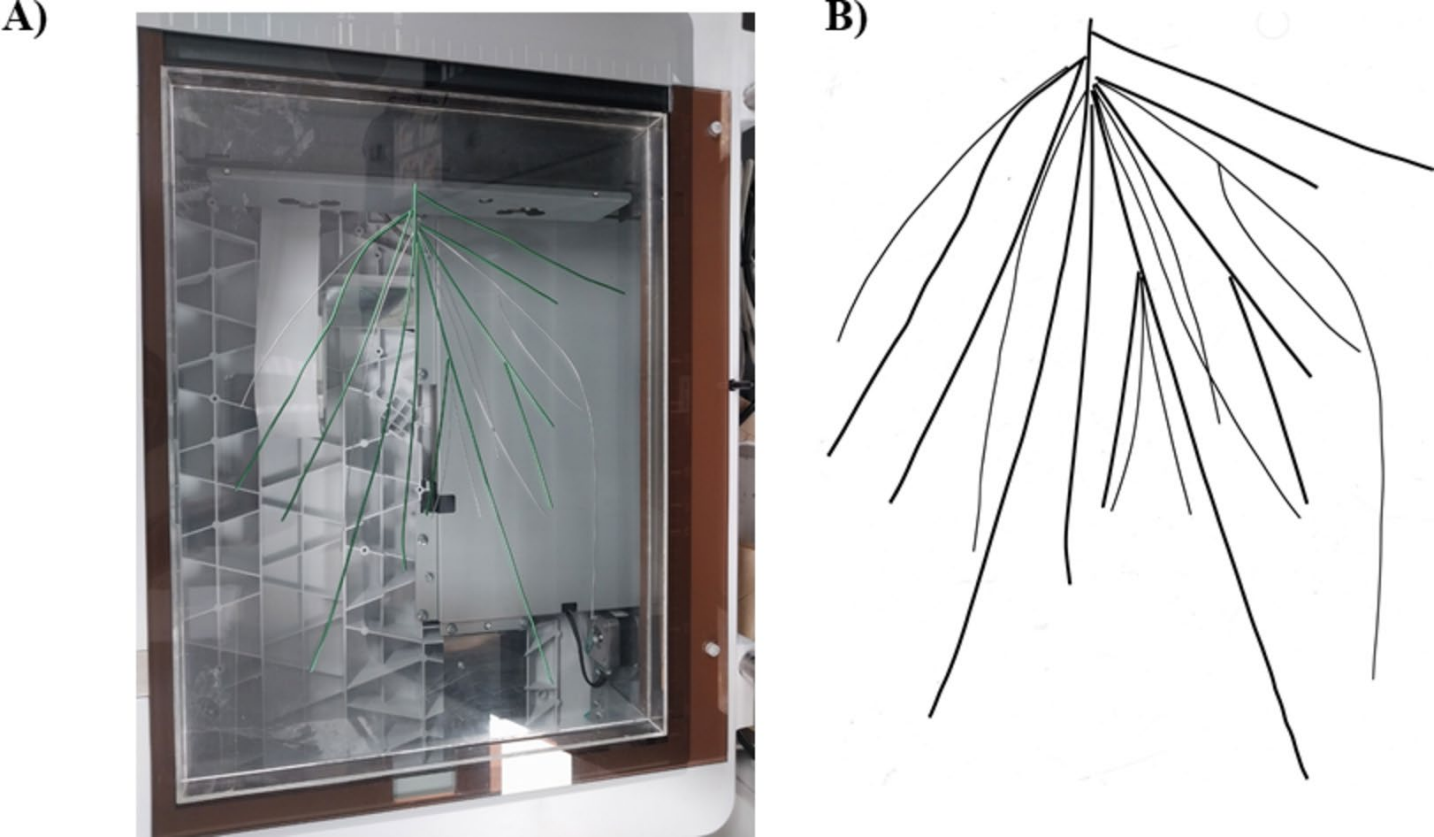
Images for validation of the algorithm. (**A**) Wires arranged on a tray on scanner, and (**B**) image obtained after scanning.

To validate the algorithm, we analyzed ground-truth data. Wires of known diameters and lengths were arranged on a scanning tray and scanned like that of the root (Fig. 3). Thus, the obtained image parameters were then analyzed with the algorithm and the two software. Furthermore, we also analyzed the ground truth images provided by Rose and Lobet (2018) and compared the results^40^ (10.5281/zenodo.1159846). A total of 100 ground truth images of 400 and 600 dpi were compared.

### Statistical analysis

The coefficient of correlation (R^2^) with a 1:1 aspect ratio was computed and visualized using RStudio 2023.03.0 Build 386. This comparison aimed to assess the results derived from the algorithm against those obtained from the software. Root mean square error (RMSE) and mean bias error (MBE) were also calculated through R-studio using ‘*Metrics*’ package.

RMSE =$$\:\sqrt{\frac{1}{n}\sum\:_{i=1}^{n}\left(oi\:-\:si\:\right)^2}$$ ---------------------- (xi)

MBE = $$\:\frac{1}{n}{\sum\:}_{i=1}^{n}\left(oi-si\right)$$ ---------------------- (xii)

where ‘*n’* is the total number of observations, ‘*oi*’ is the observed value for the i^th^ observation, ‘*si’* is the standard value for the i^th^ observation.

## Results

### Need of code development for root trait analysis

WinRHIZO stands out among various root analysis software packages due to its recognized accuracy^41,42^. This software enables the analysis of numerous root traits, including root SA, TRL, number of tips, forks, root angle, RV, AD, and number of lateral roots. However, it comes with a substantial cost. SA, TRL, AD, and RV are pivotal traits due to their close association with water and nutrient uptake and resistance to adverse soil conditions, such as drought and waterlogging^33,43^.

### Validation of the algorithm and calibration

**Fig. 4:**
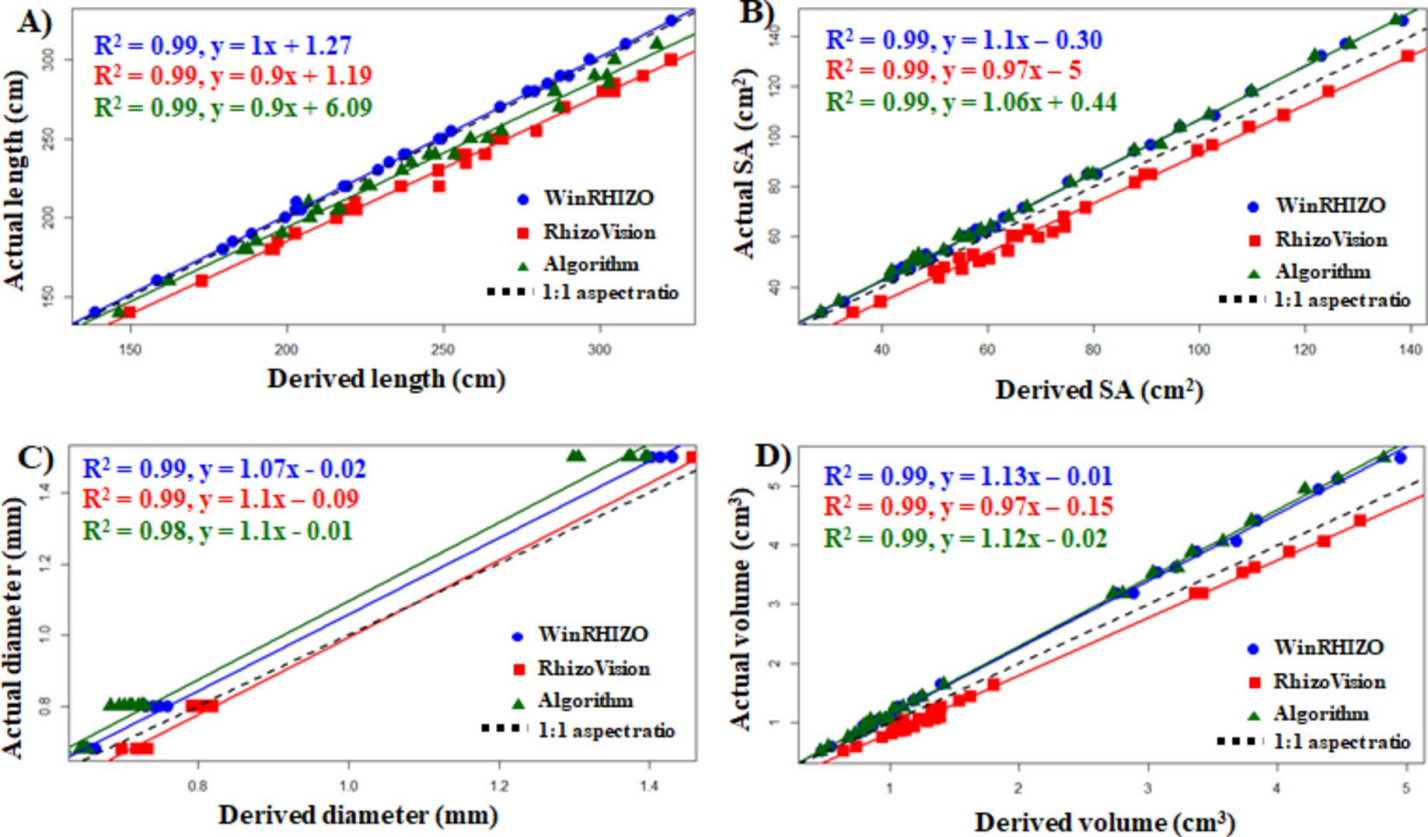
Fit of plot for algorithm-derived values against actual values. (**A**) Length, (**B**) surface area, (**C**) diameter, and (**D**) volume.

The algorithm showed a high correlation (R² ≥ 0.98, *p* < 0.001) with the ground truth value for all the traits (length, SA, diameter, and volume). Similarly, both software tools also had a strong correlation with ground truth values (R² ≥ 0.99) (Fig. 4). This high correlation means good conformity between the actual value and the software-derived values. To generalize the results obtained from different methods and compare it briefly mean values of the traits (MTV) was also calculated. All the three methods of evaluation of root traits showed a similar mean value compared with the actual mean values (**Table S2**). More deviation in mean value of total length was observed for RhizoVision, however the AD mean value of RhizoVision was exactly same to the ground-truth data (**Table S2**). To better understand the analyzed values, we also calculated the RMSE and MBE. WinRHIZO outperformed RhizoVision and the algorithm method in length and SA estimation with the least RMSE and MBE (Table 2). Here, the negative MBE indicates the underestimation, and the positive MBE indicates the overestimation of the mean values. The highest RMSE and MBE were observed in RhizoVision in both of these traits. However, in the two other traits of AD and volume, the least error values were observed in RhizoVision. The algorithm performed well in all the traits with error values similar to that of the WinRHIZO for SA, AD, and volume estimation. Likewise, the results of simulated root images also showed a good correlation and lower error values when a comparison was made between the algorithm-derived values and the actual values (Table 3). All the traits were highly correlated with the simulated root images. Length, SA and volume had R^2^ ≥ 0.98 whereas AD had the least (0.79). However, the RMSE and MBE were least in all the algorithm-derived traits compared to the simulated root images.

Likewise, to compare the calibration result we measured the total number of pixels present in between the distance of 1 cm in the scanned ruler. It was observed that 158 pixels were present in between the 1 cm span. This gave the single pixel size to be 0.006329 cm (1 / 158). The pixel size obtained from the calibration nearly matched with the pixel size obtained based on image resolution i.e., 0.0063 ≈ 0.006329.

**Table 2:** Error-values compared to ground truth images.

Methods	Traits	RMSE	MBE
WinRHIZO	Length	2.17	−1.74
	SA	5.01	−4.47
	AD	0.06	−0.05
	Volume	0.31	−0.24
RhizoVision	Length	19.22	18.6
	SA	6.96	6.65
	AD	0.03	0.006
	Volume	0.22	0.21
Algorithm	Length	9.91	8.48
	SA	5.32	4.88
	AD	0.09	−0.08
	Volume	0.34	−0.26

**Table 3:** Error-values compared to simulated root images.

Traits	RMSE	MBE	*R*-Squared
**Length**	0.33	0.05	0.99
**SA**	0.083	−0.003	0.99
**AD**	0.018	−0.002	0.79
**Volume**	0.002	0.001	0.98

### Selection of suitable thresholding methods

One crucial step in image processing is the thresholding of the image, and selecting the right threshold is essential for specific root traits. We opted for four automatic thresholding methods: Otsu, Gaussian adaptive, mean adaptive, and triangle thresholds. Twenty random roots of each legume were subjected to analysis using these thresholds. The SA, RV, and AD were based on the radius calculated through each skeletal pixel (distance transform method). We checked the results for AD only for selecting suitable thresholding methods since the AD (calculated based on the radius obtained) directly affects the other two traits, SA and RV (**Equation iv to vi**). We assessed the results both quantitively **(Table S3)** and qualitatively (Table 4). For quantitative evaluation, MBE was calculated so that the overestimation and the underestimation of the traits could be known. It was observed from the quantitative results that in the case of AD, the mean adaptive and Gaussian adaptive thresholds highly underestimated AD when compared to WinRHIZO-derived AD in all legumes with an average of −0.4 for all legumes (**Table S3**). Whereas the triangle threshold highly overestimated these values in all the legumes. The Otsu threshold performed well, where the least MBE was observed (MBE < 0.03).

Similarly, for TRL, both adaptive thresholds highly overestimated the values compared to WinRHIZO, the highest MBE being 551.875 and the least being 242.382 (**Table S3**). Here, the positive sign means the overestimation, and the negative sign means the underestimation of values compared to WinRHIZO. The Otsu threshold somewhat performed well compared to the adaptive thresholding methods, but better results were obtained for the triangle threshold, where the highest MBE was just − 28.287. To find out the reason for such values we visualized the images obtained from these different methods and present the result qualitatively in Table 4. From both quantitative and qualitative evaluations, we deduced that for radius or diameter estimation based on distance transform method Otsu threshold exhibited values within the range of software-derived values. Both methods of adaptive threshold underestimated the diameter, whereas the triangle threshold overestimated it. Similarly, for TRL estimation based on skeletonization, the Otsu method underestimated TRL, whereas both adaptive methods overestimated TRL. The triangle method emerged as the most suitable for TRL estimation, as detailed in Table 4, providing reasons for its selection. Hence, the Otsu threshold was used for distance transform, and the triangle threshold was used for skeletonization.

**Table 4:** Qualitative results for the selection of suitable thresholding methods.

Methods	Qualitative results		Remarks and reasons
	Radius/diameter based on distance transform	Skeletonization (thinning to a single pixel size)	
Otsu			For distance-transformed images, almost all root parts are visible; thus, the diameter was within the range of software-derived values. In the case of skeletonization, most of the root parts got omitted after thinning, resulting in lower TRL compared with software-derived values.
Triangle			A high threshold merged the nearby root pixels, causing a higher estimation of the diameter. Later, in the case of skeletonization, when thinning was performed, this higher intensity of the pixels caused the root parts to be well preserved, also making the TRL on the range comparable to software-derived values.
Mean adaptive			The distance-transformed image, when visualized, gave a root image where the inner skeleton was omitted, which might have caused a reduction in the radius calculation. In this method of thinning, the root image displayed a contour-like boundary structure instead of a single-pixel thinned segment; thus, counting these boundary pixels might have caused a higher estimation of the TRL.
Gaussian adaptive			The same case of mean adaptive was also observed in the Gaussian adaptive threshold, where the radius estimated was greatly reduced, whereas the TRL estimated was above the range.

### Fit of the plot

#### Fit of the plot for TRL

**Fig. 5:**
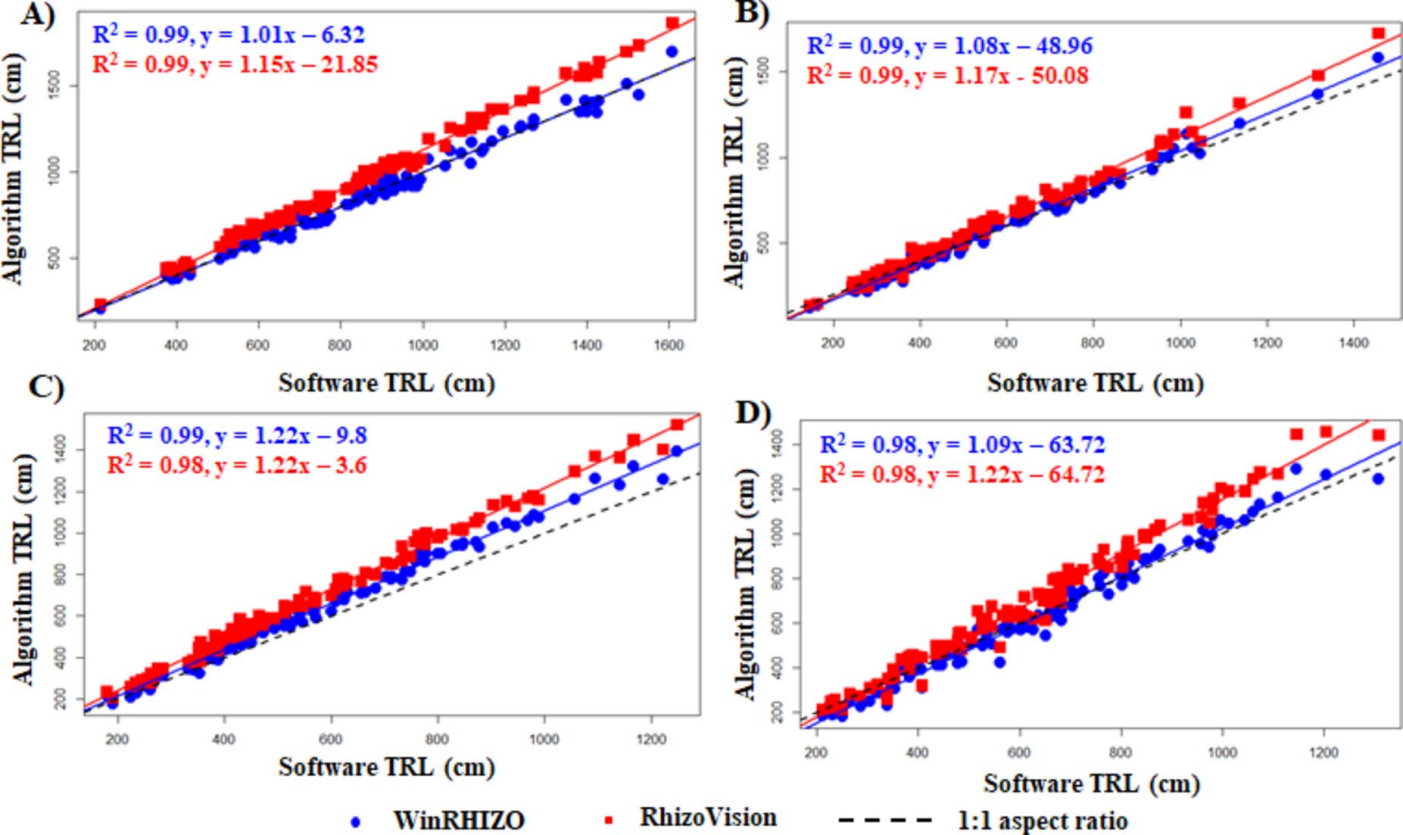
Fit of plot for algorithm-derived TRL against software-derived TRL. (**A**) adzuki bean, (**B**) mung bean, (**C**) cowpea, and (**D**) soybean.

The highest R^2^ value of 0.99 (*p < 0.001*) was observed for adzuki bean (Fig. 5A) and mung bean (Fig. 5B) when algorithm-derived TRL was compared to software-derived TRL. Similarly, R^2^ ≥ 0.98 was observed for cowpea (Fig. 5C) and soybean (Fig. 5D) for both WinRHIZO- and RhizoVision-derived TRLs against algorithm-derived TRLs. These elevated R^2^ values indicate a good conformity between the algorithm-derived TRL and the software-derived TRL. This means that the algorithm-derived TRL closely matches the TRL obtained from the software, indicating that the algorithm is reliable and produces results that are consistent with those generated by the established software.

#### Fit of the plot for SA

**Fig. 6:**
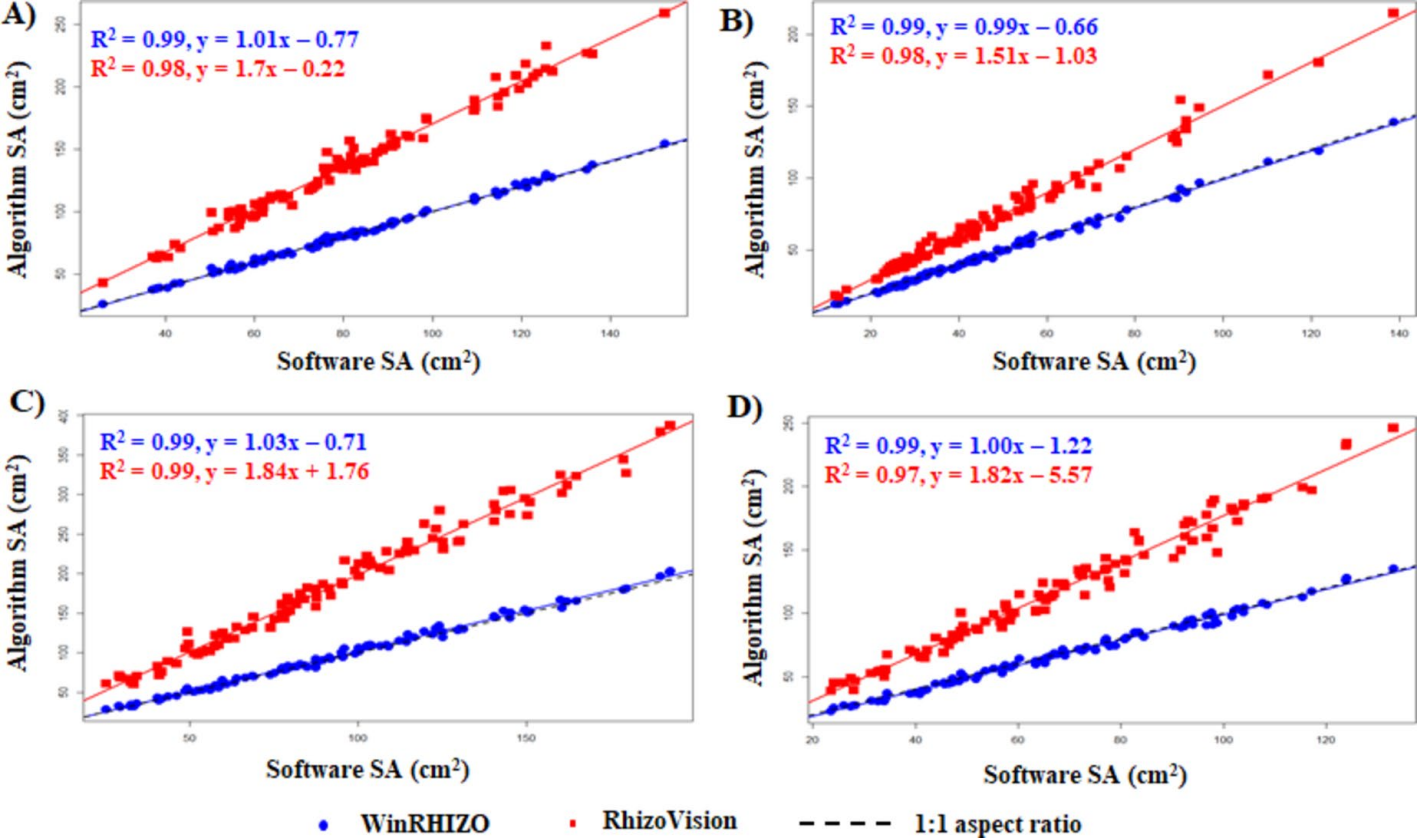
Fit of the plot for algorithm-derived SA against software-derived. (**A**) adzuki bean, (**B**) mung bean, (**C**) cowpea, and (**D**) soybean.

The plot for SA revealed that the algorithm-derived SA are highly concentrated within the regression line. An R^2^ value of ≥ 0.97 was observed in all legumes when compared with both software-derived values (Fig. 6). The SA results were more inclined towards the WinRHIZO-derived results as the 1:1 aspect ratio also coincided with the WinRHIZO’s regression line in all the legumes. The slope of the line was found to be close to 1 when compared to the WinRHIZO-derived SA, indicating strong agreement with WinRHIZO.

#### Fit of the plot for AD

**Fig. 7:**
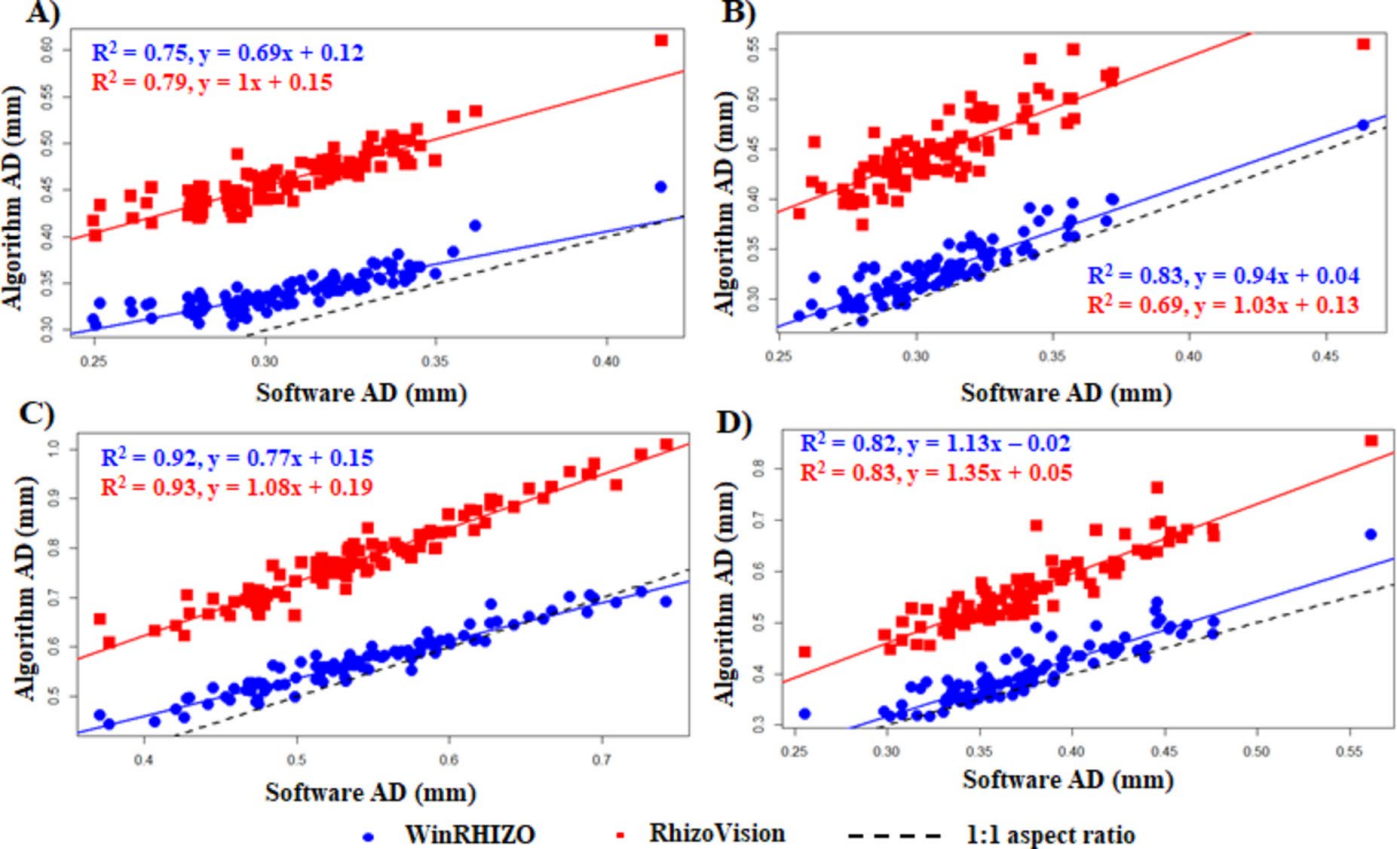
Fit of the plot for algorithm-derived diameter (AD) against software-derived AD. (**A**) adzuki bean; (**B**) Mung bean; (**C**) Cowpea; and (**D**) Soybean.

The data values for AD exhibited somewhat scattered distribution when compared with other root traits, with a wider margin. Adzuki bean (Fig. 7A), cowpea (Fig. 7C), and soybean (Fig. 7D) exhibited higher R^2^ value when compared to RhizoVision than the WinRHIZO, the highest being 0.93 for cowpea. Least correlation coefficient (R^2^ = 0.69) was observed for mung bean (Fig. 7B) when compared to RhizoVision. The algorithm-derived AD had more deviation compared to other root traits and the algorithm-derived AD was more correlated to RhizoVision than the WinRHIZO, except for mung bean.

#### Fit of the plot for RV

**Fig. 8:**
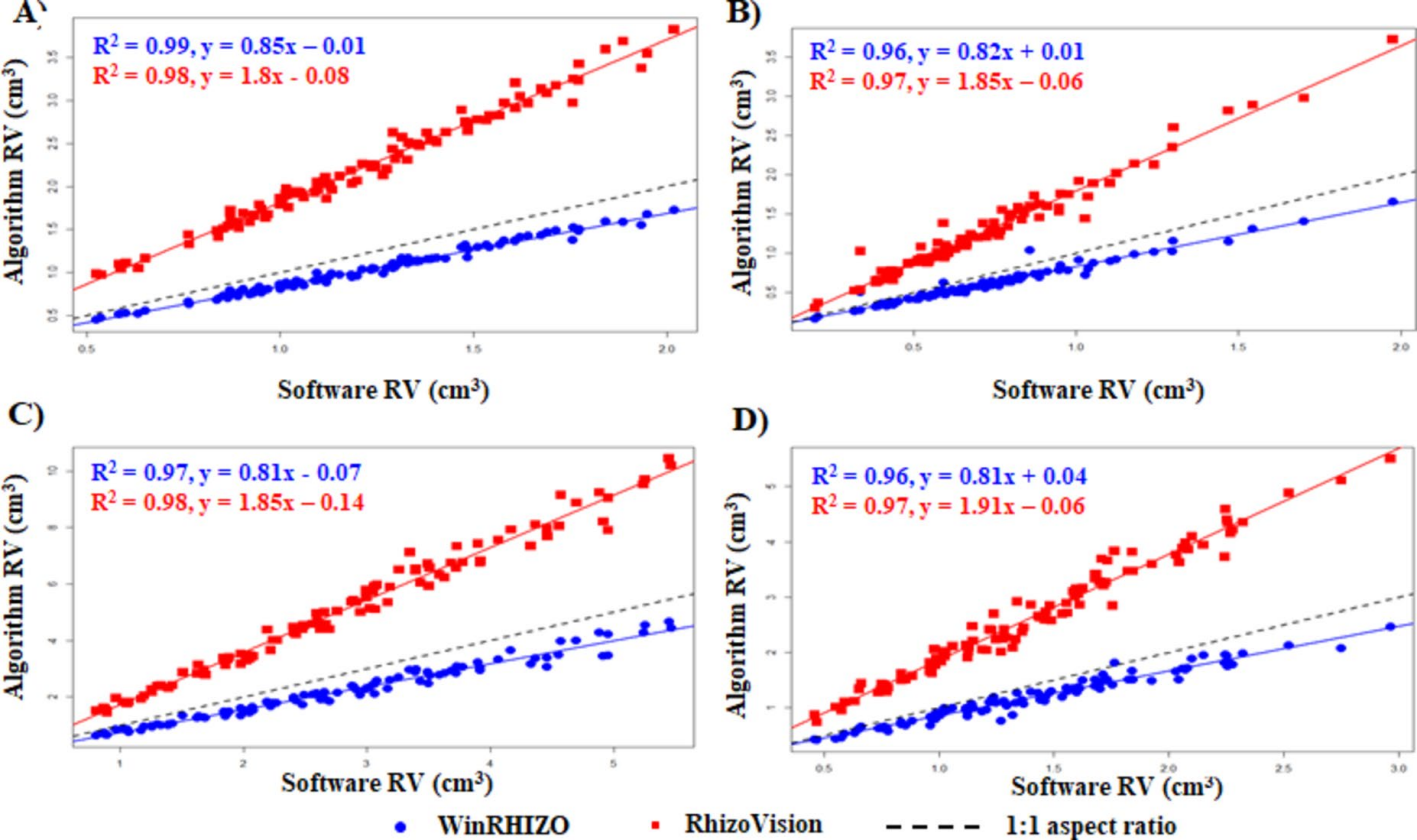
Fit of the plot for algorithm-derived RV against software-derived RV. (**A**) adzuki bean, (**B**) mung bean, (**C**) cowpea, and (**D**) soybean.

In the case of RV, the algorithm-derived RV exhibited a stronger correlation with both RhizoVision and WinRHIZO. The R^2^ value was ≥ 0.97 for RhizoVision in all legumes, with the highest observed for adzuki bean (Fig. 8A) and cowpea (Fig. 8C), with a value of 0.98. When compared to WinRHIZO adzuki bean (Fig. 8A) had the highest correlation among all the other legumes (R^2^ = 0.99). Likewise, mung bean (Fig. 8B), cowpea **(**Fig. 8C), and soybean (Fig. 8D) had R^2^ ≥ 0.96. RV overall had stronger correlation along with higher R^2^ value with both software-derived RV. This higher correlation was observed in almost all the legumes.

### Error value calculation

To compare the algorithm-derived root traits and software-derived root traits more comprehensively, we calculated the mean value for each trait, RMSE, and MBE. For the generalization of the root traits mean values were calculated (**Table S4**). In all the root traits estimated by algorithm they were closer to the WinRHIZO-derived mean values. RhizoVision derived root traits were deviated compared to the algorithm-derived root traits (**Table S4**). Table 5 presents the RMSE and MBE for the algorithm-estimated root traits compared with WinRHIZO measured values and RhizoVision software-derived root traits. It was observed that the algorithm-derived root traits had higher error values in all the legumes among all the traits when compared to RhizoVision values. The results were more inclined to the WinRHIZO-derived values. The highest RMSE of 135.08 was observed in cowpea TRL compared to RhizoVision, which was almost double the value of RMSE in WinRHIZO (70.54). The algorithm-derived TRL highly underestimated the TRL compared to RhizoVision with MBE of −106.692 for adzuki bean and − 121.912 for cowpea. However, these values were reduced when a comparison was made to WinRHIZO, where the MBE dropped to 3.5 for adzuki bean and − 58.148 for cowpea. A similar result was observed in SA estimation, where the values for RMSE and MBE were greatly increased when the algorithm-derived SA was compared to RhizoVision-derived SA. As in TRL estimation, cowpea had higher rates of errors for SA compared to other legumes with RMSE of 3.71 and MBE of −1.70 when algorithm was compared to WinRHIZO. These values increased to 88.91 and − 81.35 when the algorithm was compared to RhizoVision. Mung bean had the least error values when compared with both software-derived values. the error values of AD and RV also showed similar pattern of results like in SA. Algorithm-derived AD and RV had less error when compared to WinRHIZO whereas these error values increased when algorithm-derived AD and RV were compared to RhizoVision. In every instance, cowpea showed higher RMSE and MBE values, while mung bean consistently had the lowest values for almost all the root traits.

Furthermore, we also compared the estimated SA and RV derived from the algorithm with the WinRHIZO estimated value (**Table S5**). It was observed that these two traits derived from the algorithm were highly correlated with WinRHIZO-derived values with R^2^ ≥ 0.98, except for soybean RV. For RV the RMSE was **≤** 1.5 and MBE **≤** 0.005. For SA, the RMSE was ≤ 10 and MBE ≤ 1 (**Table S5**).

**Table 5:** Comparison of the error values of root traits derived from different algorithms with two distinct software applications.

Traits	Plants	WinRHIZO		RhizoVision	
		RMSE	MBE	RMSE	MBE
TRL	Adzuki bean	32.48	3.50	117.99	−106.69
	Mung bean	33.96	3.17	68.22	−42.35
	Cowpea	70.54	−58.15	135.08	−121.91
	Soybean	44.33	6.24	100.67	−73.45
SA	Adzuki bean	1.63	−0.22	59.43	−56.25
	Mung bean	1.58	0.75	26.67	−23.41
	Cowpea	3.74	−1.70	88.91	−81.35
	Soybean	2.52	1.00	55.66	−50.99
AD	Adzuki bean	0.04	−0.03	0.16	−0.15
	Mung bean	0.02	−0.02	0.14	−0.14
	Cowpea	0.04	−0.03	0.24	−0.24
	Soybean	0.04	−0.03	0.19	−0.19
RV	Adzuki bean	0.19	0.19	1.05	−1.00
	Mung bean	0.14	0.12	0.61	−0.54
	Cowpea	0.65	0.58	2.48	−2.22
	Soybean	0.26	0.22	1.33	−1.22

### Credibility of the study

In terms of study credibility, our algorithm underwent validation. Additionally, we provide illustrations of root structures of various sizes along with their predicted values and values derived from both WinRHIZO and RhizoVision. Two roots images, representing small and large structures, were randomly chosen from each legume, based on their characteristics (Table 6). Notably, the algorithm-derived root traits closely matched the WinRHIZO-derived values in all cases.

**Table 6.**
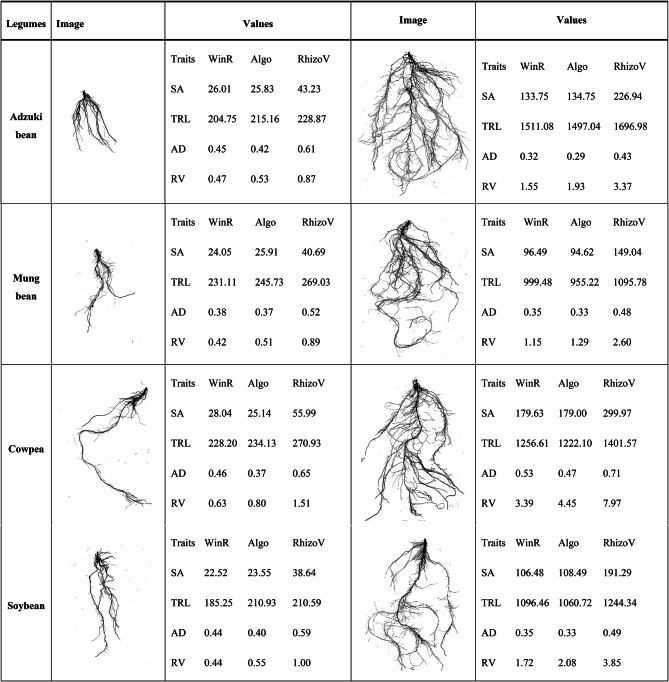
Comparative analysis of the algorithm across different root structures.

Note: WinR means WinRHIZO-derived values, Algo means algorithm-derived values, and RhizoV means RhizoVision-derived values.

## Discussion

SA, TRL, AD, and RV are important root traits with significant roles in plant growth, development, and overall crop yield. In this study, we employed a straightforward Python algorithm to estimate these traits in four different legumes. Functioning within a Python environment, the algorithm is OS-independent and does not necessitate additional software such as ImageJ or RStudio for analysis. Incorporation of the triangle, adaptive, and Otsu methods in the algorithm automates the root image thresholding process, eliminating the need for manual intervention. The triangle method is used to determine the threshold value by standardizing the height and dynamic range of the histogram of pixel intensities in an image^36^. In addition, the thinning algorithm (ximgproc thinning) automates the image-thinning process based on neighboring pixels until a single pixel is obtained. The algorithm is adaptable, allowing users to modify it according to their specific needs. The analysis does not demand advanced computer devices. Furthermore, the algorithm calculates connected components of the root parts, enabling users to set thresholds for particle clearance background noise removal. As convolution neural network (CNN) and deep learning segmentation procedures might pose challenges for agricultural researchers, our simple thresholding and thinning algorithm facilitates easy root image analysis. Users can input root images in various formats, such as .jpeg, .JPG, .png, and .TIF. With its user-friendly interface and accuracy comparable to the expensive WinRHIZO software, this algorithm set can be used by researchers without incurring any expenses. The algorithm-derived method would be useful for researcher who require to measure major root traits, as they are held back by the high cost of the existing software. Furthermore, any one working on root traits estimation might find the paper useful as detailed methodology has been mentioned for each of the traits measured. They can modify the source code and use it as per their requirement or add up more traits.

The results indicate that Otsu thresholding method produced better results for the distance transform method in estimating SA, AD, and RV. In contrast, the triangle threshold in TRL estimation yielded superior results. The Otsu threshold also showed relatively good results in TRL estimation. Similar observations were made in the estimation of root length, where the choice of threshold significantly influenced the results, and the Otsu method did not perform well in total root length estimation^36^. Notably, this study demonstrated a high correlation coefficient of 0.986 for TRL in rice when comparing ImageJ- and WinRHIZO-derived values, particularly when utilizing the triangle method of thresholding with a correction factor of 2/3. In case of saRIA, a study on Arabidopsis roots reported R^2^values of 0.8612 for SA, 0.8496 for TRL estimation, and 0.7768 for RV estimation compared with manual SmartRoot image segmentation^44^. Moreover, a comparison between saRIA and fully automated root image analysis (faRIA) resulted in R^2^values of 0.986 for SA, 0.979 for TRL, and 0.986 for RV^45^. In another study, a high correlation coefficient of 0.997 was observed between IJ_Rhizo and WinRHIZO on test images for length determination^46^. The study also reported that the smallest disparities in length measurements occurred when comparing the corrected estimates of IJ_Rhizo with Tennant values from WinRHIZO. On average, there was a 10% difference, with 25% of the samples exhibiting variances exceeding 10% from each other. In addition, the same study found an R^2^ value of 0.893 for the comparison between IJ_Rhizo and WinRHIZO AD, with an average error of 18%.

In the present study, WinRHIZO software was considered a more standard reference, although results were also compared with RhizoVision. However, it is essential to note that the results obtained from WinRHIZO may not be 100% accurate. Discrepancies of 2.9% in root SA have been observed between Tennant’s method and the WinRHIZO method of area calculation, with noted issues in measuring other root traits of fine roots^47^. As outlined in the methodology, roots were immersed in water before scanning, and it has been reported that immersing soybean roots in water before scanning significantly increased the estimated value of root SA by 20% when analyzed in WinRHIZO software^48^. Similarly, some concerns regarding the calculation of specific root length and root tissue density have been raised for WinRHIZO software by^49^. When comparing the trait root tissue density with Archimedes’ method and the image analysis method by WinRHIZO, the predicted R^2^was 0.56, indicating a relatively low correlation^50^. Similarly, in our ground truth result the RhizoVision outperformed WinRHIZO and algorithm in AD and volume estimation based on RMSE and MBE values although the difference in these errors was less. RhizoVision uses the distance transform value associated with each skeletal pixel which represents its radius, and then it is doubled to determine its diameter whereas WinRHIZO uses punctual diameter method where the smallest distance between two boundary pixels gives the diameter. Two improvements have been made in WinRHIZO for punctual diameter calculation where first one concerns about the angle at which the diameter is estimated and the second one concerns about using more intensity information from the original image. However, regarding the RV determination, contrasting results have been found among different research between the two software. Pang et al., (2011) found that WinRHIZO performed well for RV detection compared to water displacement method^51^, whereas other research by Seethepalli et al., (2021) found that when compared to ground truth value of volume, WinRHIZO had lower agreement^39^. However, it is essential to note that while using values from WinRHIZO, the measured values should be given more importance than the estimated values^49^. Thus, it is essential to conduct thorough research to consolidate all existing root phenotyping software onto a unified platform. This will enable the quantification of a more extensive dataset of ground truth values. Although this study provides a detailed methodology for measuring root traits, it only quantifies four major traits, which presents a limitation. Furthermore, modification of the source code needs to be done so that it can be applicable to very fine roots having plants like monocots.

## Conclusion

The four major root traits SA, TRL, AD, and RV were estimated in this study using four different leguminous crops at the early growth stage with an automated thresholding and thinning algorithm. Our results were validated with ground-truth data and correlated with widely used software, including WinRHIZO and RhizoVision Explorer. Consistently high accuracy was observed in almost all instances for algorithm-derived traits when compared with ground truth data and two different software. For now, users with limited resources can use this method to quantify root traits in legumes and estimate the key root characteristics. However, future works needs to be done to add up more traits and make the study applicable to other plants. An integrated phenotyping platform for evaluating all the root and shoot traits can be the best alternative.

## Electronic supplementary material

Below is the link to the electronic supplementary material.

Supplementary Material 1

## Data Availability

All the root images and images for the validation can be downloaded from (https://github.com/AG9843/Legume-Root-Analysis.git ).
