# Supplementary material for: Python algorithm package for automated Estimation of major legume root traits using two dimensional images
Source: Sci Rep. 2025 Mar 1;15:7341. doi: 10.1038/s41598-025-91993-y (PMC11873191; doi:10.1038/s41598-025-91993-y)
Supplement: Supplementary file 1 — Supplementary Material 1 [file 41598_2025_91993_MOESM1_ESM.pdf]

Python algorithm package for automated estimation of major legume root traits using two dimensional images

Amit Ghimire, Yong Suk Chung, Sungmoon Jeong, Yoonha Kim *

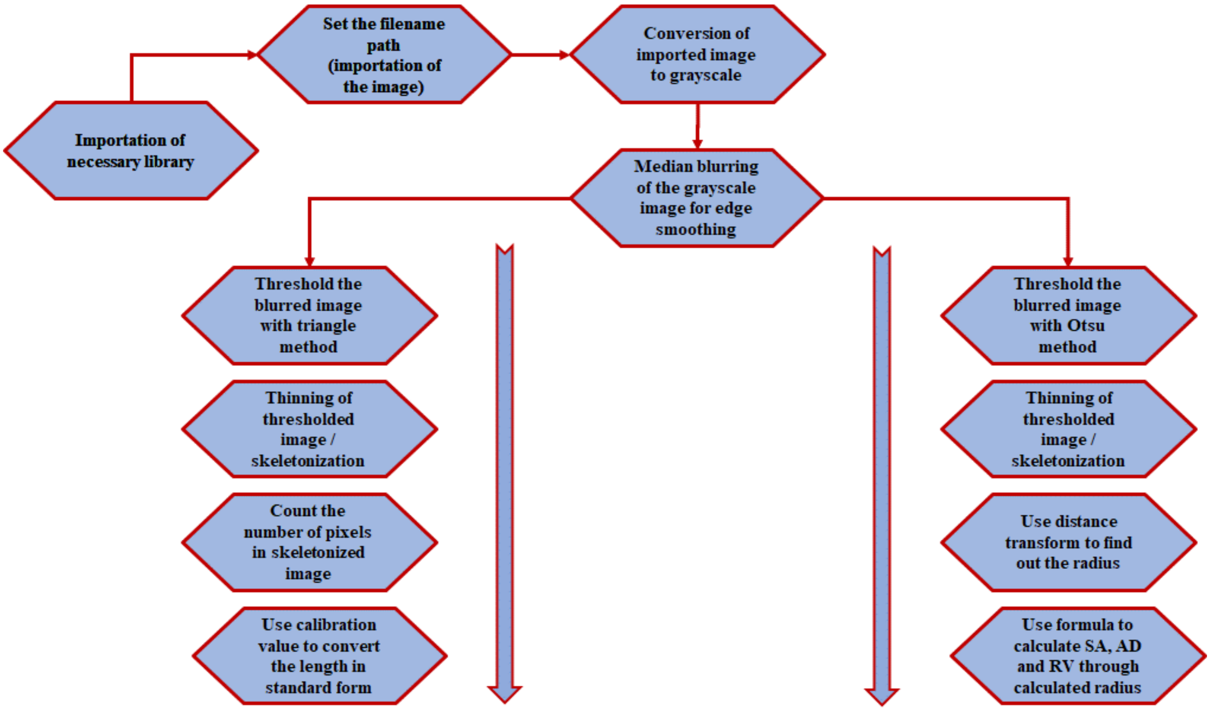

Fig. S1. Flowchart of the methodology for root trait estimation.

Table S1. Metadata features kept while analyzing the roots from RhizoVision Explorer and WinRHIZO.

**RhizoVision Explorer**	**Set parameters**	**WinRHIZO**	**Set parameters**
Root type	Broken roots	Root detection based on	Gray level
Image Thresholding Level	205	Threshold selection	Automatic
Invert images	False	Root	Darker
Keep largest component	True	Speed, Memory, Feedbacks	Medium, More, Max
Filter noisy components on background	True	Lasso resolution	Medium
Maximum background noisy component size	8	Root morphology	True
Filter noisy components on foreground	False	Precision	Standard [Fast]
Maximum foreground noisy component size	1	Link analysis	True
Enable edge smoothing	False	Topology	True
Edge smoothing threshold	2	Magnitude	Standard
Enable root pruning	True	Developmental	True
Root pruning threshold	5	Order continuation criteria	Magnitude [+]<br>Branching angle [-]<br>Diameter [+]
Convert pixels to physical units	True		
Dots per inch	400		

Pixel to millimeter conversion factor	0.0635
Diameter Range 1	0
Diameter Range 2	2
Diameter Range 3	5

Table S2. Mean values of the traits obtained for ground-truth data.

Traits	Methods	Values
Length	Actual	235.67
	WinRHIZO	233.93
	RhizoVision	254.27
	Algorithm	244.14
SA	Actual	73.52
	WinRHIZO	69.04
	RhizoVision	80.17
	Algorithm	68.63
AD	Actual	0.99
	WinRHIZO	0.94
	RhizoVision	0.99
	Algorithm	0.91
Volume	Actual	2.06
	WinRHIZO	1.83
	RhizoVision	2.27
	Algorithm	1.80

Table S3. MBE error among different thresholding methods for AD and TRL estimation.

Thresholding methods	Adzuki bean	Mung bean	Soybean	Cowpea
	AD MBE			
Otsu	0.002	0.027	0.030	0.009
Triangle	0.394	0.390	0.456	0.771
Gaussian adaptive	-0.332	-0.322	-0.380	-0.519
Mean adaptive	-0.332	-0.322	-0.379	-0.517
	TRL MBE			
Otsu	-89.372	-46.928	-65.789	-126.718
Triangle	-14.764	26.227	5.334	-28.287
Gaussian adaptive	525.100	344.671	551.875	242.382
Mean adaptive	461.406	321.184	498.999	193.102

Table S4. Mean values of the traits obtained for different legumes.

Traits	Plants	Mean trait values (MTV)		
		WinRHIZO	Algorithm	RhizoVision
TRL	Adzuki bean	853.13	856.69	963.37
	Mung bean	531.04	534.21	576.56
	Cowpea	630.16	572.01	693.92

	Soybean	624.42	631.56	705.01
SA	Adzuki bean	79.99	79.77	136.01
	Mung bean	46.74	47.49	70.90
	Cowpea	95.90	94.20	175.55
	Soybean	67.63	68.64	119.63
AD	Adzuki bean	0.34	0.31	0.46
	Mung bean	0.33	0.31	0.45
	Cowpea	0.57	0.54	0.78
	Soybean	0.40	0.38	0.56
RV	Adzuki bean	1.02	1.21	2.21
	Mung bean	0.59	0.70	1.24
	Cowpea	2.18	2.76	4.99
	Soybean	1.18	1.40	2.63

Table S5. Comparison of WinRHIZO estimated root traits with algorithm estimated root traits.

**Legumes**	**Traits**	**RMSE**	**MBE**	**R-squared**
Adzuki bean	SA	3.27	0.61	0.99
	RV	0.05	0.005	0.98
Mung bean	SA	1.71	0.068	0.99
	RV	0.03	-0.003	0.98
Cowpea	SA	10	-8.15	0.99
	RV	0.15	-0.089	0.99
Soybean	SA	6.18	-4.82	0.99
	RV	0.11	-0.093	0.95
